# DNA Pooling Base Genome-Wide Association Study Identifies Variants at NRXN3 Associated with Delayed Encephalopathy after Acute Carbon Monoxide Poisoning

**DOI:** 10.1371/journal.pone.0079159

**Published:** 2013-11-12

**Authors:** Wenqiang Li, Yanxia Zhang, Renjun Gu, Ping Zhang, Fei Liang, Jiapeng Gu, Xuemin Zhang, Hongya Zhang, Hongxing Zhang

**Affiliations:** 1 Department of Neurology, the Second Affiliated Hospital of Xinxiang Medical University, Xinxiang, China; 2 Henan Key Lab of Biological Psychiatry, Xinxiang Medical University, Xinxiang, China; 3 Health Team of the 93123 Unit, The Chinese People’s Liberation Army, Dalian, China; 4 Yang Pu District Center for Disease Control and Prevention, Shanghai, China; 5 Tongzhou Hospital for Matenal and Child Health Care, Beijing, China; Yale School of Public Health, United States of America

## Abstract

Delayed encephalopathy after acute carbon monoxide poisoning (DEACMP) is more characteristic of anoxic encephalopathy than of other types of anoxia. Those who have the same poisoning degree and are of similar age and gender have a greater risk of getting DEACMP. This has made it clear that there are obvious personal differences. Genetic factors may play a very important role. The authors performed a genome-wide association study involving pooling of DNA obtained from 175 patients and 244 matched acute carbon monoxide poisoning without delayed encephalopathy controls. The Illumina HumanHap 660 Chip array was used for DNA pools. Allele frequencies of all SNPs were compared between delayed encephalopathy after acute carbon monoxide poisoning and control groups and ranked. A total of 123 SNPs gave an OR >1.4. Of these, 46 mapped in or close to known genes. Forty-eight SNPs located in 19 genes were associated with DEACMP after correction for 5% FDR in the genome-wide association of pooled DNA. Two SNPs (rs11845632 and rs2196447) locate in the Neurexin 3 gene were selected for individual genotyping in all samples and another cohort consisted of 234 and 271 controls. There were significant differences in the genotype and allele frequencies of rs11845632 and rs2196447 between the DEACMP group and controls group (all *P*-values <0.05). This study describes a positive association between Neurexin 3 and controls in the Han Chinese population, and provides genetic evidence to support the susceptibility of DEACMP, which may be the resulting interaction of environmental and genetic factors.

## Introduction

Acute carbon monoxide (CO) intoxication is not uncommon in the world at present [1]–[2]. Most surviving patients can recover completely after acute CO intoxication; however, 0.2–40% of survivors develops serious delayed encephalopathy within 2–6 weeks after this pseudorecovery [3]–[4]. Because of this, delayed encephalopathy is more characteristic of anoxic encephalopathy than of other types of anoxia; therefore, it was named delayed encephalopathy after acute carbon monoxide poisoning (DEACMP).

DEACMP is clinically characterized by a recurrence of neurologic or psychiatric symptoms, and involves a triad of characteristic symptoms consisting of mental deterioration, urinary incontinence, and gait disturbance [5]. Brain magnetic resonance imaging (MRI) revealed multiple lesions in the subcortical white matter and basal ganglia, mostly in the globus pallidus, and a lesser degree in the putamen, and caudate. The patients remain in a state of cognitive dysfunction with extrapyramidal damage after DEACMP. A history of cardiocerebrovascular disease, intoxication time and age were risk factors for DEACMP [6].

The mechanisms of DEACMP in humans and in experimental animals are not fully understood [7]–[8]. Our previous studies have demonstrated significant differences of neuron-specific enolase, myelin basic protein, interleukin and other immune cytokines in serum and cerebrospinal fluid in the development of DEACMP [9]–[12], which suggested that DEACMP may be the outcome of acute-carbon-monoxide-poisoning-induced brain immune damage and apoptosis. The efficiency of repair after central nervous system injury is related to susceptibility to DEACMP. However, the occurrence varied greatly, even in patients with similar age, sex and intoxication level, which suggested that individual differences exist, and that genetic factors might play an important role. In our previous report, the case-control study showed that there was an association between the MBP TGGAn gene polymorphism and DEACMP [13].

Genome-wide association studies (GWAS) have been successful in the identification of loci contributing to complex diseases. However, a limitation of GWAS is the large number of hypotheses tested and the high economic cost [14]. Many studies have addressed the feasibility and effectiveness of pooling-based genome-wide association (GWA) with considerable savings in time and costs [14]–[16]. In this study, we carried out a GWAS using DNA pools of cases and controls constructed separately for men and women to allow the identification of a susceptibility gene. The result was duplicated in an additional cohort of individual samples.

## Materials and Methods

### Subjects

The protocol was approved by the Ethical Committee of the Second Affiliated Hospital of Xinxiang Medical University. Written informed consent was obtained from all participants after the objectives and procedures of the study were fully explained. If a patient was unable to give informed consent, it was signed by his/her caregiver. All study protocols were approved by the Institutional Review Board of the Second Affiliated Hospital of Xinxiang Medical University.

The DEACMP group consisted of patients who experienced delayed encephalopathy after acute carbon monoxide poisoning. The acute carbon monoxide poisoning (ACMP) group was followed-up for more than 90 days, and no DEACMP was observed. Clinical and demographic characteristics are shown in Table 1. Patients in the DEACMP group were regarded as cases and patients in the ACMP group as controls. All participants were unrelated Han Chinese born individuals living in the North Henan province, and all of their biological grandparents were of Han Chinese ancestry. The age, sex and education level were matched between these two groups. Peripheral blood samples from each subject were drawn into vacutainer tubes containing the anticoagulant ethylenediaminetetraacetic acid (EDTA); this was performed in DECAMP patients from 6∶00 am to 8∶00 am, and in acute CO poisoning patients within 2 h after fully conscious recovery. All blood samples were stored at −70°C. The genomic DNA was extracted from peripheral blood leucocytes using standard protocols.

**Table 1:** Summary of the demographic and clinical data from the cohorts included in the study.

Variable	DEACMP		ACMP	
	Test sample (n = 175)	Replication sample (n = 234)	Test sample (n = 244)	Replication sample (n = 271)
Age [mean±S.D.]	59.68±9.72	54.14±10.51	56.12±7.38	55.47±9.34
Gender				
Males (n)	84	115	104	164
Females (n)	91	119	144	107
Education				
illiterates	54	74	77	91
primary school	63	95	82	97
middle or high school	58	65	85	83
Coma time (h)	16.09±15.13	14.24±12.68	15.22±15.13	17.55±14.68

### Pooling GWA

In the first stage, we conducted GWA analysis using a DNA-pooling approach. Genomic DNA was extracted from peripheral blood leukocytes using the RelaxGene Blood DNA System (Tiangen Biotech, Beijing, China). DNA samples were verified for integrity by agarose gel electrophoresis and samples with 260/280 absorbance ratios <1.8 were extracted with phenol/chloroform again. Once the DNA samples were ready for the pooling experiments, DNA concentrations were measured using spectroscopy (260 nm) and samples were diluted to 50 ng/µl. Each sample to be pooled was subsequently quantified using the fluorometer (Invitrogen, Carlsbad, USA). If necessary, the DNA concentration was adjusted [14]–[16]. DNA pools were constructed by combining equimolar amounts of 100 ng DNA from each participant. Four sets of pooled samples were created: group A was the female DEACMP group (*n* = 91), group B was the male DEACMP group (*n* = 84), group C was the female acute carbon monoxide poisoning (ACMP) group (*n* = 140), and group D was the male ACMP group (*n* = 104). Illumina HumanHap 660 Whole-Genome Genotyping BeadChip was performed for DNA pooling. To assess variance in allele frequency attributable to the pooling procedure, each pool was created third and the technical replicate pools were compared. Twelve chips (each pool was replicated in triplicate) were used according to the manufacturer’s instructions. Standard quality control was applied.

### Analysis of Pooling Data

The raw allele frequency data of the DNA pools were derived from the Illumina BeadStation software. The correction factors were derived for each SNP from raw intensity data from individuals assayed on the genotyping platform. DNA from a heterozygous individual is equivalent to DNA from a pool with a 50% allele frequency. So the correction factor *k* for each SNP were calculated based on the average ratio of dye intensities (A: B) across all known heterozygotes [14], [17]. The mean *k* value was 0.87. The *k* value were applied to the relative allele signal (RAS = A/(A+B)) for each SNP to get a raw allele frequency [RAFk = A/(A+k*B)] [14]. For each SNP, extreme values were normalized to data obtained from the RAS values of known homozygotes [14]. The RAF of each SNP was averaged across replicate pools. SNP with a variance between replicate pools of >5% were excluded.

We calculated normalized allele frequencies from raw intensity data and averaged data across replicate pools to obtain a relative allele frequency (RAF) estimate for each SNP in each pool. SNP-pool combinations with a variance between replicate pools of >2% were excluded. RAFs were compared using the 1 df chi-square test between two groups in same gender. Multiple testing was controlled using a false discovery rate (FDR) at 5%. SNPs were ranked based on logarithm (base 10) of the P-value (logP) by the gender. The sum was calculated for the order in the two genders. The SNP with smaller sum get high rank. When the sum is same, the SNP with the bigger difference of RAF between two groups is prior.

### SNP Selection for Individual Genotyping

Forty-eight SNPs were selected by the following criteria. SNPs that were high-ranked in both genders and located in known genes were included in the panel. They were all tagSNPs using data from the International HapMap project (www.hapmap.org; CHB+JPT population) [18]; their characteristics are shown Table 2. Two SNPs, rs11845632 (A/G) and rs2196447 (A/G), located in the Neurexin 3 (NRXN3) gene, were selected to be individually genotyped in this study.

**Table 2:** Top 10 SNPs associated with DEACMP in the pooling-based GWA in both genders.

	SNP	Chromosome	Position	*P* value		Gene
				Female	Male	
1	rs11845632	14	79023567	1.27×10^−8^	1.49×10^−7^	Neurexin 3
2	rs3747869	10	73190638	1.34×10^−7^	1.22×10^−8^	Cadherin-related 23
3	rs1368387	5	147876501	1.84×10^−8^	5.33×10^−7^	5-hydroxytryptamine (serotonin) receptor 4
4	rs11550299	11	66010661	4.04×10^−7^	1.53×10^−7^	Dipeptidyl-peptidase 3
5	rs6028103	20	59319547	4.27×10^−7^	5.09×10^−7^	Cadherin 4, type 1, R-cadherin (retinal)
6	rs2196447	14	79020241	5.30×10^−7^	2.83×10^−7^	Neurexin 3
7	rs6445588	3	53605719	6.29×10^−7^	1.97×10^−7^	Calcium channel, voltage-dependent, L type, alpha 1D subunit
8	rs4295733	9	140098397	6.84×10^−7^	5.38×10^−7^	Calcium channel, voltage-dependent, N type, alpha 1B subunit
9	rs10247883	7	9075442	7.16×10^−7^	5.66×10^−7^	Colony stimulating factor 2 receptor, beta, low-affinity
10	rs867522	5	147946439	9.01×10^−7^	5.57×10^−7^	5-hydroxytryptamine (serotonin) receptor 4

### Individual Genotyping

Initially, two SNPs were selected to be individually genotyped in all pooled samples. Another cohort that consisted of 234 DEACMP patients (115 males and 119 females) and 271 ACMP patients (164 males and 107 males) was also individually genotyped.

The two SNPs were detected by polymerase chain reaction based restriction fragment length polymorphism (PCR-RFLP) analysis. For rs11845632 (promoter region), the sequences of the primers were 5′-ATGTGCTACATGGTGAAAGGCA-3′ and 5′-TTGAAAGGAAATGCAGTTTGGTA-3′. For rs2196447 (intron 2), the sequences of the primers were 5′-GTGGGTGTCCATATGGAAGTCAC-3′ and 5′-CTCCCAGGAATGTAGGAAGGAAG-3′. The sizes of the amplified fragments were 141 bp and 243 bp, respectively. The PCR amplification was performed in a 25-µl reaction volume containing 10 × PCR buffer 2.5 µl, dNTP mix (10 mM) 0.5 µl, each primer (10 µM) 1 µl, genomic DNA 100 ng, *Taq* DNA polymerase (Tiangen Biotech, Beijing, China) 1U and sterile deionized water. After initial denaturation at 94°C for 5 min, the sample was amplified using 35 cycles of 94°C for 30 s, 60°C for 30 s, and 72°C for 60 s, followed by a final elongation at 72°C for 10 min. The PCR product was digested at 37°C for 2 h using the restriction enzymes *Rsa*I (for rs11845632) and *Cse*I (for rs2196447) (Thermo Fisher Scientific, Waltham, USA), then analyzed by electrophoresis on 2% agarose gels. The resulting images were screened and saved using the gel documentation system. Genotypes were identified by two investigators independently. The three genotypes resulting from digestion with *Rsa*I were AA (141 bp), AG (141 bp, 119 bp) and GG (119 bp) for rs11845632. Similarly, the three genotypes yielded by digestion with *Cse*I were GG (243 bp), AG (243 bp, 183 bp) and AA (183 bp).

### Statistical Analysis

For individual genotyping results, deviation from Hardy-Weinberg equilibrium was examined in the control population for each SNP using the chi-square test. Associations between alleles and genotypes and DEACMP risk were evaluated using the Pearson chi-square test and Cochran-Armitage trend test, respectively. Per-allele odds ratios (ORs) and corresponding 95% confidence intervals (95% CIs) were also calculated for each SNP. Power analysis was performed using the Genetic Power Calculator [14], [19]. All statistical analysis was two-tailed, and the level of statistical significance was defined at adjusted *P*<0.05.

## Results

A genome-wide analysis was performed with three pools of DNA from DEACMP patients and ACMP patients. Population characteristics are shown in Table 1. In total, 842 SNPs differed in frequency between cases and controls at *P*<0.05 and 123 SNPs had an OR >1.4. Of these, 46 mapped in or close to known genes. Forty-eight SNPs were associated with DEACMP after correction for 5% FDR in the GWA of pooled DNA; these SNPs were located in 19 genes. The top 10 P-value SNPs are shown in Table 2.

From these results, one gene was selected for individual genotyping at a second stage which presented two SNPs (rs11845632 and rs2196447) associated with DEACMP in the pooled analysis. This gene is Neurexin 3 (NRXN3), which is a member of the neurexin family of proteins that function in the vertebrate nervous system as cell adhesion molecules and receptors. The SNP rs11845632 is a splice site SNP that is located 23 bp from the splice site in intron 4.

The individual genotyping results are shown in Table 3. The differences in allele frequencies between cases and controls were similar in the pooled and individual data (mean absolute difference was 0.102 for rs11845632 and 0.081 for rs2196447). SNPs remained significantly associated with DEACMP at the *P*<0.05 level when genotyped individually in the sample. The association was replicated in another cohort consisting of 234 DEACMP patients and 271 ACMP patients. Power analysis revealed that the pooling sample size (n = 419) had 80% power to detect a (r >0.37) effects on genotype distributions, the total sample size (n = 924) had 97% power.

**Table 3:** Individual genotyping results for the two SNPs of Neurexin 3.

Variable		Test sample		Replication sample		HWE(*p*)*	*P-* values*		*OR(95% CI)* *
		DEACMP% (n = 175)	ACMP% (n = 244)	DEACMP% (n = 234)	ACMP% (n = 271)		Genotype	Allele	
rs11845632	AA	18(10.2)	46(18.9)	28(11.9)	48(17.7)	0.339	0.007	0.0002	1.00
	AG	87(49.7)	124(50.8)	108(46.1)	138(58.9)				1.42 (1.07–1.89)
	GG	70(40.1)	74(30.3)	98(42)	85(23.4)				2.16 (1.43–3.27)
rs2196447	AA	27(15.4)	25(10.2)	40(17.1)	32(11.8)	0.071	0.006	0.0002	1.00
	AG	96(54.9)	116(47.5)	124(52.9)	129(47.6)				0.64 (0.48–0.85)
	GG	52(29.7)	103(42.3)	70(30.0)	110(40.6)				0.49 (0.32–0.74)

* For combined samples.

Significant differences were found in genotype and allele frequencies between patients and controls for rs11845632 and rs2196447 (*P*<0.05 for all) in pooled samples. The frequency of the rs11845632 G allele was greater in DEACMP patients (64.9%) than in ACMP patients (56.3%, OR = 1.44, 95% CI = 1.19–1.73) in combined samples, and the frequency of the rs2196447 A allele was greater in DEACMP patients (43.2%) than in ACMP patients (34.9%, OR = 1.43, 95% CI = 1.18–1.72; 34.0%).

## Discussion

DNA pooling has been confirmed to be an effective and efficient method to select candidate susceptibility loci for follow-up by individual genotyping [15]–[16], [20]. In this study, we performed a two-stage DNA pooling-based GWAS for DEACMP, evaluating a genome-wide panel of markers in two subject groups using a DNA pooling strategy; in the second stage we replicated two promising SNPs at an individual level in the individual pooled samples as well as in a second set of cases and controls. In total, 842 SNPs showed a significant difference in frequency between cases and controls. We identified NRXN3 as a potential gene associated with DEACMP.

DEACMP is a common and still poorly explained organic mental disorder [21]–[22]. The pathological hallmark is extensive demyelination, and current theories for the pathogenic mechanism include a direct toxicity effect of CO, cerebral blood vessel damage, and cerebral edema [23]. During the initial anoxic insult, no clinical signs distinguish patients who will develop delayed neurological sequelae, but a triggering role of early ambulation or emotional stress has been postulated [24]. Age and severity of the initial anoxia increase the risk of late encephalopathy, but this is not always the case [5]. For some psychiatric disorders after brain injury, the genetic predisposition is another important element, as is brain structure damage. Some individuals would be susceptible, as those who have a genetic predisposition would have a lower threshold for the emergence of symptoms when they are exposed to environmental risk factors (hypoxia, traumatic brain injury) or even during their normal neurodevelopment. Starkstein and colleagues [25] suggested that the genetic predisposition for mood disorders and focal lesions in relative brain areas may both provide the factors necessary for the development of mania symptoms after traumatic brain injury. Schwarzbold et al. [26] considered psychosis associated with traumatic brain injury to have a multifactorial etiology, whose genetic factors have a non-Mendelian nature. A single nucleotide polymorphism of the KIBRA gene was reportedly associated with episodic memory in individuals with severe traumatic brain injury [27]. Polymorphisms of the serotonin transporter gene were identified as risk factors for post-stroke depression, as well as other kinds of organic mental disorder [28]. The present study provides evidence that genetic polymorphism is associated with DEACMP. These data suggest the genetic susceptibility of organic mental disorders.

NRXN3, which is located on chromosome 14q31, encodes a member of the neurexin family, which shows high expression in the central nervous system. Neurexins are cell adhesion molecules that help to specify and stabilize synapses and provide receptors for neuroligins, neurexophilins, and dystroglycans [29]. Genetic variation in cell adhesion molecules are thought to cause individual differences in vulnerability to human neuropsychiatric disorder [30]. Individual differences in cell adhesion molecule expression could lead to differences in the development of brain circuits and adult brain circuits, as well as differences in the adaptation of the brain to environmental factors. NRXN3 has previously been reported to play an important role in mental disorders and behavior [31], and is a compelling biological candidate for obesity [32]–[33]. Data from postmortem human cerebral cortical brain samples showed that NRXN3 polymorphisms are associated with alcohol dependence and the altered expression of specific isoform [30]. The genome-wide linkage study of opioid dependence located a region on chromosome 14q; the peak encompasses the NRXN3 gene [34]. A recent study observed an association between NRXN3 polymorphisms and borderline personality disorder phenotypes in heroin-dependent cases [35]. The association of NRXN3 polymorphisms with schizophrenia was also found in a large case-control study [36]. The results of Novak et al. [37] suggest that variants in the NRXN3 gene could contribute to the degree of nicotine dependence in patients with schizophrenia, while another association study confirmed a role for NRXN3 in susceptibility to smoking behavior [38]. As an organic mental disorder, DEACMP shows psychotic symptoms including visual and auditory hallucinations, persecutory delusions, depression and cognitive impairments [39]. The meta-analysis of five GWAS identified a marker in the NRXN3 gene (rs17757879) that showed a consistent protective effect in Alzheimer’s disease [40]. The association with DEACMP enhances NRXN3 as a susceptibility gene for mental disorders and neurological disease.

There is a discrepancy between the pooled p-values and the p-values for the individually genotyped SNPs. Despite significant replication values of some promising SNPs found in the GWA in pooled DNA, many papers show the lack of replication for some of the loci identified in the pooling based analysis. Some loci lost the statistical differences in individually stage [41]–[42]. Even the loci got replication, some the pooled p-values and the p-values for the individually had a disparity [43]–[45]. There are several possible explanations for the above discrepancies.

The first possibility relates to the different sample size for pool construction and replication. The discrepancy of p-values is a common feature in two stages case-control studies. For smaller sample size, the margin of sampling error is larger, the possibility of variation in allele frequency is larger. The second possibility relates to the different of data analysis. It’s impossible to detect and adjust all potential confounders that could produce the inconsistencies in the analysis of DNA pooling. DNA pooling analysis depends on RAF, system errors likely enlarger. The third possibility relates to technology. Compared to individual genotyping, DNA pooling does add extra experimental error (e.g. due to errors in pool construction) to the allele frequency measurements. For DNA pooling, the method for measure allele frequency differences were also affected by the number of subjects included in pool. So the DNA pooling focuses initial screening of promising SNPs, a two-stage design in which the most promising SNPs identified in the screening stage are followed up by individual genotyping, like us in the current study. The results were replicated in the individual and more samples and replication is considered essential to establish the validity of finding.

## Conclusions

In summary, we have found suggestive evidence for an association between DEACMP and specific SNPs (rs11845632 and rs2196447) in the NRXN3 gene. Our research has shown that there was genetic susceptibility to DEACMP, and that DEACMP was a disease resulting from interactions between environmental factors and an individual’s genetic background. Independent replications and functional analysis are necessary to fully confirm this locus as a genuine susceptibility gene in DEACMP. More valuable discoveries would be expected through expansions of the sample size and further study.
